# Supplementary material for: Role and mechanism of NCAPD3 in promoting malignant behaviors in gastric cancer
Source: Front Pharmacol. 2024 Apr 22;15:1341039. doi: 10.3389/fphar.2024.1341039 (PMC11070777; doi:10.3389/fphar.2024.1341039)
Supplement: Supplementary file 11 [file DataSheet2.ZIP › GSEA/Transcipt factor/my_analysis.Gsea.1599461814734/FOXO1_01.html]

Details for gene set FOXO1\_01[GSEA]

|  || Dataset | filtered\_dataset.sample\_info.cls#WT\_versus\_NCAPD3\_MUT |
| Phenotype | sample\_info.cls#WT\_versus\_NCAPD3\_MUT |
| Upregulated in class | NCAPD3\_MUT |
| GeneSet | FOXO1\_01 |
| Enrichment Score (ES) | -0.45854697 |
| Normalized Enrichment Score (NES) | -2.1335926 |
| Nominal p-value | 0.0 |
| FDR q-value | 0.03992848 |
| FWER p-Value | 0.268 |
Table: GSEA Results Summary

  

Fig 1: Enrichment plot: FOXO1\_01      
 Profile of the Running ES Score & Positions of GeneSet Members on the Rank Ordered List

  

| SYMBOL | TITLE | RANK IN GENE LIST | RANK METRIC SCORE | RUNNING ES | CORE ENRICHMENT || 1 | 7248 | TSC1 | 158 | 0.718 | -0.0557 | No |
| 2 | 9075 | CLDN2 | 420 | 0.522 | -0.2013 | No |
| 3 | 1979 | EIF4EBP2 | 532 | 0.456 | -0.2442 | No |
| 4 | 4784 | NFIX | 567 | 0.439 | -0.2333 | No |
| 5 | 84335 | AKT1S1 | 572 | 0.437 | -0.2009 | No |
| 6 | 26100 | WIPI2 | 601 | 0.423 | -0.1869 | No |
| 7 | 7067 | THRA | 976 | -0.392 | -0.4243 | Yes |
| 8 | 901 | CCNG2 | 998 | -0.405 | -0.4067 | Yes |
| 9 | 53349 | ZFYVE1 | 1018 | -0.417 | -0.3866 | Yes |
| 10 | 2012 | EMP1 | 1119 | -0.494 | -0.4187 | Yes |
| 11 | 54541 | DDIT4 | 1133 | -0.502 | -0.3875 | Yes |
| 12 | 639 | PRDM1 | 1159 | -0.523 | -0.3632 | Yes |
| 13 | 27250 | PDCD4 | 1170 | -0.537 | -0.3270 | Yes |
| 14 | 2119 | ETV5 | 1176 | -0.545 | -0.2866 | Yes |
| 15 | 54407 | SLC38A2 | 1182 | -0.553 | -0.2455 | Yes |
| 16 | 153222 | CREBRF | 1187 | -0.559 | -0.2032 | Yes |
| 17 | 861 | RUNX1 | 1293 | -0.687 | -0.2233 | Yes |
| 18 | 3084 | NRG1 | 1320 | -0.739 | -0.1823 | Yes |
| 19 | 10397 | NDRG1 | 1380 | -0.925 | -0.1501 | Yes |
| 20 | 55729 | ATF7IP | 1392 | -1.034 | -0.0744 | Yes |
| 21 | 26959 | HBP1 | 1395 | -1.073 | 0.0108 | Yes |
Table: GSEA details [plain text format]

  

Fig 2: FOXO1\_01      
 Blue-Pink O' Gram in the Space of the Analyzed GeneSet

  

Fig 3: FOXO1\_01: Random ES distribution      
 Gene set null distribution of ES for **FOXO1\_01**
